# ERRATUM: The use of the Nursing Activities Score in clinical settings: an integrative review

**DOI:** 10.1590/1980-220X-REEUSP-2024-ER20en

**Published:** 2024-09-30

In the article “The use of the Nursing Activities Score in clinical settings: an integrative review”, with the DOI: https://doi.org/10.1590/S0080-623420150000700021, published by the journal: “Revista da Escola de Enfermagem da USP, volume 49 (spe) of 2015, p. 147–156, on page 156:

**Where it was written:**

## Financial support:

 this study was funded through a subvention provided by Réseau de Recherche en Interventions en Sciences Infirmières du Québec (RRISIQ).

**Now read:**

## Financial support

This study was funded through a subvention provided by Réseau de Recherche en Interventions en Sciences Infirmières du Québec (RRISIQ). This study was financed in part by the Coordenação de Aperfeiçoamento de Pessoal de Nível Superior - Brasil (CAPES) - Finance Code 001.

